# Negative Refractive Index Metasurfaces for Enhanced Biosensing

**DOI:** 10.3390/ma4010001

**Published:** 2010-12-23

**Authors:** Zoran Jakšić, Slobodan Vuković, Jovan Matovic, Dragan Tanasković

**Affiliations:** 1 Center of Microelectronic Technologies and Single Crystals (CMTM), Institute of Chemistry, Technology and Metallurgy (IHTM), University of Belgrade, Njegoševa 12, 11000 Belgrade, Serbia; E-Mail: dragant@nanosys.ihtm.bg.ac.rs (D.T.); 2 Institute of Physics, University of Belgrade, 11080 Zemun, Serbia; E-Mail: svukovic@ipb.ac.rs (S.V.); 3 Institute for Sensor and Actuator Systems (ISAS), Faculty of Electrical Engineering & Information Technology, Vienna University of Technology, Gusshausstrasse 27-29/366-MST, A-1040 Vienna, Austria; E-Mail: Jovan.Matovic@tuwien.ac.at (J.M.)

**Keywords:** plasmonics, optical metamaterials, artificial nanomembranes, long range surface plasmons polaritons, chemical sensors, biosensors

## Abstract

In this paper we review some metasurfaces with negative values of effective refractive index, as scaffolds for a new generation of surface plasmon polariton-based biological or chemical sensors. The electromagnetic properties of a metasurface may be tuned by its full immersion into analyte, or by the adsorption of a thin layer on it, both of which change its properties as a plasmonic guide. We consider various simple forms of plasmonic crystals suitable for this purpose. We start with the basic case of a freestanding, electromagnetically symmetrical plasmonic slab and analyze different ultrathin, multilayer structures, to finally consider some two-dimensional “wallpaper” geometries like split ring resonator arrays and fishnet structures. A part of the text is dedicated to the possibility of multifunctionalization where a metasurface structure is simultaneously utilized both for sensing and for selectivity enhancement. Finally we give an overview of surface-bound intrinsic electromagnetic noise phenomena that limits the ultimate performance of a metasurfaces sensor.

## 1. Introduction

The demand for various types of chemical, biochemical or biological (CBB) sensors is constantly increasing [1]. A CBB sensor may be generally described as a device which generates a readable output (most often electrical or optical) proportional to the amount of a targeted analyte in a given environment. Various uses in biomedicine, process industry, forensics, homeland defense and other fields pose demands which include high sensitivity and selectivity, fast response, low noise, possibility of massively parallel multisensor operation and low cost [2]. Among the convenient devices an important role belongs to surface plasmon polariton (SPP) sensors [3].

An SPP sensor is actually a simple waveguide for surface plasmon polaritons, planar waves supported at sensor-environment interface and evanescent in both directions perpendicular to it. The sensor surface is a metal slab with a negative value of relative dielectric permittivity, while the dielectric permittivity of the ambient is positive. When analyte is adsorbed at the sensor surface (either directly or through a target-specific ligand layer) or is brought into contact with it in some other way, for instance by full immersion, it modifies the refractive index “seen” by the SPP wave and in this manner modulates the propagating conditions. The SPP wave must use some kind of a coupler with the propagating plane waves used for excitation and readout, because its wavevector is typically much larger than that of the propagating waves. This is done using some external means, for instance a refractive prism in Kretschmann [4] or Otto configuration [5], optical grating, surface corrugations or some other coupling device [6]. A SPP chemical or biological sensor is label-free, contrary to many of other competing types of CBB sensors and, owing to its electromagnetic nature, sufficiently fast to ensure measurements in real time.

A recently proposed generalization of the SPP sensor involves the use of electromagnetic metamaterials (MM) instead of simple metal surfaces. A prototype example of a metamaterial is a composite structure with the real part of its effective refractive index being less than zero (“negative refractive index—NIM” materials, also sometimes denoted as “left-handed” metamaterials or “double-negative” structures) [7,8,9]. Although the first metamaterials described were NIM structures, the term is nowadays used in a broader sense to denote various synthetic composite structures with properties not readily found in nature. These may also include (but are not limited to) the so-called single negative materials, the artificial structures in which only relative dielectric permittivity or only relative magnetic permeability reaches values lower than zero (ENG—Epsilon Negative or MNG—Mu Negative), as well as various other structures with “unusual” properties, an example being 2D arrays of subwavelength apertures with extraordinary optical transmission [10].

Many novel electromagnetic effects appear in the MM, some of which may appear counter-intuitive (opposite directions of phase velocity on one side, and group velocity and Poynting vector on the other side, reversal of the Snell’s law, *etc*.) [11]. An obvious thought is to combine such properties, many of which could be used to enhance freedom in sensor design, with the already existing benefits of the conventional SPP sensors. In this way one uses electromagnetic metamaterials to obtain generalized plasmonic sensors of chemical, biochemical and biological analytes [12,13].

It is usually considered that metamaterials are ridden with problems which seriously impede their practical applicability. Among the greatest of these disadvantages are their large spectral dispersions with resonant shapes and sharp peaks, as well as their high absorption losses. Although these properties do represent an obstacle to many real-life implementations, the situation is reversed for sensing purposes; sharp peaks and resonances in response are actually beneficial here. On the other hand, absorption losses are mostly not an issue for sensing. This can be seen from the fact that there is large absorption in metal of conventional surface plasmon resonance sensors and their application is nevertheless wide and expanding.

Another property of MM is a strong localization and enhancement of fields. Like in the SPP devices, these can be used to actually improve the sensor selectivity when nonlinear substances are detected and to enable detection of extremely small amounts of analytes.

Finally, since optical MM actually represent nanocomposites, their structure can in principle be optimized to maximum tuning by a specific analyte. For instance, one could tailor resonances to coincide with those of a specific analyte and in that manner simultaneously obtain high sensitivity and high selectivity. Theoretically, one could design a desired response function of a MM CBB sensor from scratch.

The field of metamaterial-based CBB sensors is only at its beginning stage, metamaterials themselves being introduced only about a decade ago, while the first metamaterial-based sensors were proposed only a few years ago. An important hindrance to earlier introduction of metamaterial-based plasmonic sensors was the complex nanofabrication of metamaterials for the optical range. A way used to circumvent this obstacle is the use of planar structures, the so-called metasurfaces, also denoted as “wallpaper” metamaterials. Such structures require conventional planar and micro/nanosystem technologies for their fabrication and are much easier to make than any 3D composite. This is the reason why practically all metamaterial sensors introduced until now have been based on metasurfaces.

An optical metasurface may be considered a 1D or 2D surface plasmon polariton waveguide—a subwavelength plasmonic crystal (SPC) [14]. In certain modes such structures may be considered the optical counterparts of transmission line metamaterials [15]. The simplest metasurfaces are 1D plasmonic crystals—actually representing laminar stacks of ultrathin films with alternating strata of materials with different values of refractive indices, typically metals and dielectrics. A more complex case uses 2D subwavelength plasmonic crystals (“wallpaper” structures) which include various 2D periodic metal-dielectric composites.

In this review, we overview some possible approaches to metasurface-based sensors of chemical, biochemical and biological analytes. One should mention, however, that here we do not consider imaging sensors based on metamaterials [16,17]. We focus strictly on adsorption-desorption and immersion-based (fluidic) chemical or biological sensors with metasurfaces as active areas.

The review is organized as follows. After giving a short overview of experimental works with different types of electromagnetic metamaterials, we consider a novel scaffold for planar metamaterial-based sensors, the ultrathin freestanding self-supported films (the nanomembranes), which ensure full electromagnetic symmetry for metasurfaces. In the main part of the text we deal with single-negative magnetic metamaterials (split ring resonators), electric metamaterials (laminar ultrathin planar structures), and double-negative structures (fishnet media). Further we investigate the possibility of utilizing the same metasurface simultaneously for sensing and for selectivity enhancement, we then consider intrinsic sources of noise which limit the ultimate performance of metamaterial-based CBB sensors, and finally draw some conclusions.

## 2. An Experimental Outlook to Metamaterial Fabrication and Sensing Applications

Different definitions of metamaterials are found in literature. Cai and Shalaev define them as “artificial functional materials engineered to fulfill the prescribed electromagnetic properties” that are “beyond conventional materials” [9], while Pendry and Smith describe them as artificial structures with “properties unlike any naturally occurring substance” [16]. In this text we use the definition that currently appears to be most widespread, that metamaterials are synthetic composites with subwavelength details which furnish properties not readily found in nature.

Most of the researchers agree that metamaterials attain their properties from the unit structure instead of the constituent materials, while that unit structure has subwavelength dimensions so that their electromagnetic (optical) properties may be expressed utilizing homogenized material parameters (effective medium theory).

The term “Metamaterials” was introduced in 1999 by Rodger M. Walser [17] and was first published in literature by Smith *et al.* in 2000 [18]. As mentioned in the introduction, probably the best known metamaterials are the structures with negative effective refractive index (NIM), however the quoted definition is much more general and basically comprises all structures furnishing “unusual” electromagnetic response. Some examples of general metamaterials include two-dimensional arrays of subwavelength holes which furnish “extraordinary” high optical transmission [10,19,20,21,22,23,24], structures with near-zero effective refractive index [25,26], artificial single-negative materials (for instance, MNG metamagnetic for the optical wavelength range [27], structures with extraordinary optical gyrotropy [28], *etc*.

Despite the properties that may appear contra-intuitive, there is nothing extraordinary about metamaterials themselves and they are a natural consequence of material structuring/compositing, well explained and described by Maxwellian physics. The natural atoms/molecules in conventional materials represent an effective electromagnetic medium which the incident radiation “sees” as homogeneous. The situation is analogous with metamaterials, with the difference that we are free to create artificial electromagnetic “atoms” or “molecules”, synthetic structures—“particles” larger than natural atoms and molecules but much smaller than the operating wavelength, so that again they are seen as an effective medium by the utilized electromagnetic radiation. Further, the advent of nanotechnologies enabled us to create electromagnetic “particles” sufficiently small to ensure their use at optical, even visible frequencies (*i.e*., with nanometric characteristic dimensions).

When metamaterials were first introduced [7], they were a mere scientific curiosity and did not attract much attention. In 1999 they were exposed to the attention of the scientific community by the seminal paper of Pendry *et al*. [8]. In the beginning, they represented a controversial topic, their nature being (unsuccessfully) disputed [29,30]. Subsequently their use for sub-diffraction limited imaging (as proposed by Pendry [31]), was shortly questioned in [32]. After a huge body of experimental works has been published, it appears now that a consensus is reached in the scientific community about the nature and applicability of electromagnetic metamaterials.

The first experimental metamaterial, a hybrid consisting of split ring resonators furnishing negative effective magnetic permeability and wire mesh media with negative effective permittivity, was described by Smith *et al*. in *Science* [18]. Subsequent experiments were presented by Shelby *et al*. in *Science* [33]. Further pioneering experimental works on negative refractive index metamaterials based on split-ring resonators included [34,35,36]. Another group of experimental metamaterial structures were those based on transmission line approach and the first publications in this area include [37,38,39,40]. These structures are planar, basically represent metasurfaces and are thus of interest for this review.

Shalaev *et al.* fabricated in 2005 optical metamaterial based on gold nanorod pairs (cut wire pair metamaterial) on a planar substrate [41]. In the same year Zhang *et al.* reported a fishnet-type metal‑dielectric‑metal multilayer structure showing a negative refractive index in the near-infrared of around 2 μm [42]. In 2006 Schurig *et al.* described in *Science* an experimental metamaterial cloaking device in the microwave range [43]. Metamaterial devices with active components intended for the terahertz range were presented in *Nature* in [44], while loss-free negative index metamaterials for the optical range were reported in the same journal in 2010 [45]. Experimental NIM structures in the visible were described by Xiao *et al.* [46] (yellow part of the spectrum) and by Burgos *et al.* [47] (blue part of the spectrum).

Numerous practical applications were proposed in the meantime, some of which are quoted in [48], first for the microwave range, then for shorter wavelengths as well, and the number continues to grow to this day.

A review of progress in creating nanostructured metamaterials for optical frequencies was published by Shalaev in *Nature Photonics*. Comprehensive reviews of experimental works in the fields of optical metamaterials were written by Boltasseva and Shalaev in *Metamaterials* [49] and by Busch *et al.* in *Physics Reports* [50]. Also a number of monographs and handbooks appeared dedicated to electromagnetic metamaterials [9,11,51,52,53,54,55,56,57,58,59,60].

A consequence of the interest in optical metamaterials was a new impetus to research the general plasmonics field. This was further intensified when Ozbay published the idea to use plasmonics as a link between optical and electric circuits, proposing to bridge the gap between photonics and electronics by nanoscale plasmonic structures and devices [61]. This is also of particular interest for the metasurface-based sensors (and generally metamaterial-based and plasmonic sensors and other devices) since it ensures direct coupling of such devices with electronic circuitry, while retaining their nano-dimensions. It actually implicitly introduces a new use for metamaterial structures, since they can be used in a dual role in this scheme, not only as the active device but as the plasmonic coupler as well.

Techniques for fabrication of metamaterials reported so far are numerous and one could claim that the majority of the techniques used for micro and nanofabrication were sooner or later applied for the fabrication of general metamaterials. The electron-beam writing technique was utilized, for example, in [41]. Patterning of metamaterials useful for metasurface-based sensing was described in [62]. Nanoimprint lithography for the fabrication of plasmonic crystal sensors was presented in [63,64,65]. An important technique is focused ion beam writing, which was used for the fabrication of metamaterials in [66]. Lamination/stratification of several layers was utilized in [67,68]. Self-assembly techniques were used for example in [69,70], as well as in [71].

A procedure to extract the effective optical parameters from experimental data was first reported by Smith *et al*. [72].

One of the first mentions of the applicability of metamaterials for sensing purposes dates as far back as 2001 [73]. Theoretical proposals were published that described the use of metamaterials as conventional plasmon sensors [12] and as generalized nanoplasmonic sensors utilizing both surface and volume adsorption for chemical sensing [13]. Various experimental works with metamaterial sensors include, for example the use of nanowire-based near-zero index materials [74,75,76,77], different types of planar metamaterials based on split ring resonators [78,79,80,81,82], but also nanohole array-based chemical sensors [83,84,85,86,87,88,89].

## 3. Artificial Nanomembranes as Scaffolds for Symmetric Metasurfaces

Artificial freestanding or free-floating nanomembranes represent a very convenient platform for the fabrication of metamaterials intended for operation in the optical wavelength range. Although known in various forms for some time now, the artificial nanomembranes were relatively recently introduced as a novel building block for micro and nanosystems [90] and even more recently as a platform for plasmonics [2,91]. Throughout this text we utilize the definition of the artificial nanomembranes as synthetic structures with a thickness below 100 nm and with large lateral dimensions-to-thickness aspect ratios, starting from about a hundred and reaching 1,000,000 and more [92]. It should be noted that sometimes the same name is used to denote nanoporous membranes (pore size < 100 nm), regardless of their thickness or aspect ratio [93].

In this section, we outline some basic properties of nanomembranes, stressing those of interest for nanoplasmonics and for optical metamaterials.

Nanomembranes can be made in a variety of inorganic and organic materials, which includes the plasmonic ones [94]. The use of artificial metal-containing ultrathin nanocomposite membranes for sensors has been described in [91].

Organic membranes can be used as a support and as a dielectric part for the nanocomposite metamaterial membrane. There is a vast array of eligible macromolecular materials for that purpose. Generally, organic nanomembranes tend to be sensitive to elevated temperature, humidity, and can be destroyed by various solvents. Their Young’s modulus is usually low, the range of the operating temperatures narrow and they are subject to plastic deformations and creep under constant stress. These properties are more pronounced in nanomembranes than in bulk structures of the same material.

Inorganic ultrathin nanocomposites include metals and metal composites, various oxides, semiconductor materials, different inorganic compounds but also pure elements. Various metals were reported as freestanding nanomembrane materials, including chromium, titanium, tungsten, nickel, aluminum, silver, gold, platinum; most of these being structural metals having both electromagnetic and mechanical functions at the same time. Elemental semiconductor nanomembranes were also reported, and among them, an especially important mention belongs to silicon freestanding structures, which are connected with the most widespread and mature technology. Silicon with a thickness ranging between 10 nm and 100 nm was mentioned for instance in the context of nanomembrane-based stretchable electronics [95]. Buckled silicon nanoribbons and full nanomembranes were also reported [96].

An important material for nanomembranes in CBB sensor applications is carbon, which may be used in membranes in the form of carbon nanotubes [97] or as freestanding, ultrathin diamond or diamandoid film [97]. The excellent mechanical properties of such carbon-based materials make them convenient for their use as reinforcements for the nanometer-thin freestanding structures, but also as the dielectric part of the metasurfaces.

Other classes of inorganic freestanding nanomembranes include oxide, nitride and carbide structures, many of them used either as wide-bandgap semiconductors or insulators. Silicon dioxide nanomembranes [98] are among the important ones, again because of the widely available and mature silicon technology. Other materials include silicon nitride, titanium dioxide, gallium arsenide, *etc*.

A special class of interest for this review belongs to plasmonic materials. These include Drude metals. Freestanding gold films with a thickness below 100 nm have been known for a long time [99]. In our experiments we fabricated chromium-containing nanomembranes down to 8 nm thickness and with areas of tens of millimeters square [94,100]. Another possibility to obtain freestanding nanomembranes with plasmonic properties is to utilize non-metallic Drude materials like transparent conductive oxides (e.g., tin oxide, indium oxide, *etc.*) [101,102].

**Figure 1:**
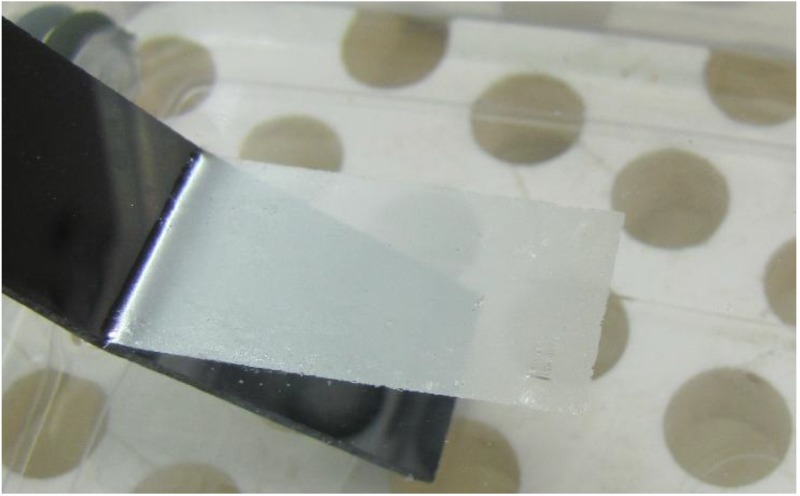
Free-floating laminar metal/dielectric/metal nanomembrane, strata thickness 10 nm + 15 nm + 10 nm, metal Au, dielectric silica, lateral dimensions 2 cm × 8 mm, support polished Si.

Symmetric plasmonic nanomembranes may be fabricated as laminar nanocomposites. Possible implementations include sandwich structures in which top and bottom layers are plasmonic material, while the middle layer may be any material serving as a support. Figure 1 shows an example of our free-floating nanomembrane with an overall thickness of 35 nm and a metal-dielectric-metal structure.

Generally, nanomembranes offer a variety of novel properties, some of which stem from their quasi-two-dimensionality and large degree of confinement of charge carriers within them. Among the properties of interest for freestanding CBB sensors is also their mechanical strength. Some types of nanomembranes are surprisingly robust on a macroscopic level and exhibit high mechanical strength with simultaneously a high flexibility and pliability. Although their thickness is in the nanometer range, it has been noted that metal-composite nanomembranes can be mechanically handled with standard lab equipment with only a moderate caution and have even been handled by bare hands [94]. Nanomembranes made of organic/inorganic interpenetrating nanocomposites were reported to be sufficiently robust to hold amounts of liquid 70,000 times heavier than their own weight, and simultaneously flexible enough to pass undamaged through a syringe with an opening 30,000 times narrower than their lateral size [90].

A very important property of freestanding nanomembranes, from the point of view of plasmonics and generally electromagnetics, is their symmetry. An identical environment surrounds them both from above and from below, being it vacuum, air or generally any fluid. If they are used as, for example, adsorption-based sensors, their symmetry still remains intact, since again the same analyte layer is present both on the top and on the bottom.

A class of plasmonic structures, where the electromagnetic symmetry may be very important to decrease absorptive losses, are the long range surface plasmon polariton guides [103,104,105]. The ultrathin freestanding membranes have been reported as especially useful platform for such guides [106]. If a plasmonic material slab between the two identical dielectric media is sufficiently thin to make the interaction between the top and the bottom SPP non-negligible, these modes couple and merge into a single one. The degeneracy for that mode is thereby removed and its dispersion is split into two branches, one odd for the low-frequency mode, and the other even for the high-frequency mode. The even modes have a very short propagation path (shorter with decreasing slab thickness), while the opposite is true for the odd modes. Their attenuation along the layer will be very low, and the propagation paths large, compared to the conventional SPP. Most of the long range modes are not contained within the plasmon part, meaning that the absorption losses will decrease. Thus long-range surface plasmon polariton-based CBB sensors are especially convenient for biological analytes, since biological samples which are typically relatively large are more easily encompassed. It has been reported that an important cause of the signal attenuation in such structures is their deviation from electromagnetic symmetry [107].

Certain types of multilayer (laminar) membrane structures of metal-dielectric type (more generally, those containing strata with positive and negative relative dielectric permittivity) actually represent plasmonic metamaterials [108,109]. Their frequency dispersion curves can be engineered by choosing the geometry of the unit cell of such a structure (strata thickness) and constitutive materials (lossy plasmonic part and dielectric part). A variety and richness of plasmonic properties is thus achievable [110,111,112,113,114,115], including those where the group velocity becomes negative [109] and therefore belong to the negative refractive index (NIM) metamaterials.

Another obvious approach to using metamaterials as sensing structures is to functionalize a freestanding or free-floating (nano)membrane by utilizing various micro- and nanofabrication technologies (as described in more detail in the next sections) in such a manner to produce a metasurface. Experimental membrane-based large-area metamaterial structures produced for sensing purposes were described in [116,117], where the authors utilized thin silicon nitride membranes as the scaffold for their terahertz-range metamaterials. Other metamaterials utilizing complementary asymmetric split-ring resonators (square unit cell, 375 nm size) produced as slits in 70 nm gold layer were fabricated on 100 nm thick freestanding silicon nitride nanomembranes in 30 × 30 μm arrays and used to obtain metamaterial electro-optic switches.

We further consider typical structures convenient for the use as CBB sensors based on planar metamaterials. The freestanding nanomembranes are appraised within this context as symmetric scaffolds for general metasurfaces.

## 4. 1D Plasmonic Crystals as Negative Index Metamaterials

The simplest case of planar plasmonic surfaces are 1D subwavelength plasmonic crystals. Such structures belong to the wider class of the so-called electric metamaterials [9] which ensure the possibility to design and tune any desired value of relative dielectric permittivity. A subclass of electric metamaterials are metal-dielectric multilayers furnishing negative value of effective relative dielectric permittivity (epsilon-negative, ENG). These structures support propagating modes and in addition to that each metal-dielectric interface in such a laminar structure will support surface plasmon polariton modes. These SPP modes become coupled through a resonance process (the coupled-resonator optical waveguide) [118], which results in the appearance of new collective modes that are confined to the interface plane and evanescent in the direction perpendicular to the multilayer surface.

A one-dimensional subwavelength plasmonic crystal (SPC) may be also considered a periodic nanocomposite lattice with alternating layers (laminated ultrathin film). These include a metal stratum (relative dielectric permittivity below zero in the operating frequency range) and a dielectric one (relative dielectric permittivity above zero). In the simplest case the structure is binary, *i.e*., its unit cell consists of a single metal and a single dielectric stratum. More complex situations include ternary, quaternary, *etc*. structures. They may consist of different materials (which actually reduces to different values of their complex relative dielectric permittivity and relative magnetic permeability, *i.e*. different complex refractive index), different values of the strata thickness, or both at the same time. In all such situations, due to the geometrical properties of the multilayers which are completely different in the direction parallel to the strata and perpendicular to them, it is obvious that the effective optical properties of such 1D SPC will be strongly anisotropic.

Plasmonic bandgaps will appear in the 1D SPC dispersion dependences, similar to the photonic bandgaps appearing in all-dielectric mesoscopic structures of photonic crystals. The behavior of 1D SPC will be actually similar to uniaxial crystal in that it will support a plethora of different modes, including surface and guided ones [115].

Depending on the geometry and dimensions, as well as on the type of terminating strata, 1D SPC multilayers guides may be symmetric, antisymmetric or asymmetric [119,120,121].

Experimentally, 1D SPC (ultrathin metal-dielectric multilayers, also known as transparent metals) are very well known and have been used for various practical application for a long time [122]. They are by far the easiest to practically implement using some of various available methods for ultrathin film deposition (most often radiofrequent sputtering and thermal evaporation) and ensure operation in frequency ranges covering infrared, visible, ultraviolet and even shorter wavelengths [123,124].

An important field of use of subwavelength metal-dielectrics within the context of metamaterials with negative refractive index are the sub-diffraction limit lenses (the so-called superlenses and hyperlenses) [16,31,125] and generally transformation optics [126]. Important experimental works utilizing subwavelength metal-dielectric multilayer-based for sub-diffraction imaging include [127,128,129,130].

Our own experiments dedicated to 1D SPC included various alternating metal-dielectric laminar structures fabricated by radiofrequent sputtering, typically using silver for the metal part and silica as dielectric [119,131]. We utilized these structures as tunable bandpass filters for the UV spectral range and used both plasmonic and propagating modes for the enhancement of their performance [131].

**Figure 2:**
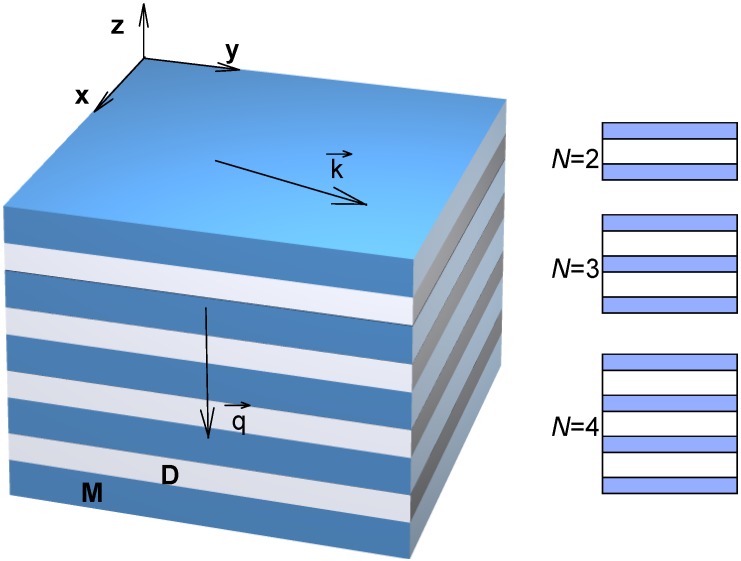
Geometry of the 1D subwavelength plasmonic crystal. M—metal; D—conventional dielectric. *N* denotes the number of cladding material strata.

We consider an electromagnetic wave propagation through a planar SPC waveguide as shown in Figure 2; $\vec{k}$ denotes the wavevector (parallel to the x-y plane, *i.e*., parallel to the strata) and $\vec{q}$ is the Bloch vector (normal to the x-y plane and parallel with the z-plane, *i.e*., perpendicular to the strata).

The unit cell is a simple binary one, consisting of a metal layer (thickness *d_m_* and a Drude-type complex relative dielectric permittivity ε*_m_* with its real part lower than zero) and a dielectric layer (thickness *d_d_* and real and dispersionless relative dielectric permittivity ε*_d_*) with a total thickness *L* = *d_d_*+ *d_d_*. The multilayer is located between thick claddings.

We consider only the symmetric situation where the top and bottom claddings are identical and are either both dielectric (vacuum or some other dielectric) or both metal. In either situation we assume that the cladding material is identical to one of the constituent materials of the plasmonic crystal. Experimentally, such a situation corresponds, for example, to [128,131].

The structure represents a waveguide with an SPC core supporting propagation of both in-plane waves (along the wavevector) and perpendicular ones (along the Bloch vector). An electromagnetic wave propagating through such a planar SPC waveguide may have TE (S) polarization or TM (P) polarization.

First we consider the case of an infinite multilayer. A well-known Floquet-Bloch dispersion is applicable to it [132,133]

(1) $$\text{cos}\left(qL\right)=\text{cos}\left(k_{m}d_{m}\right)\text{cos}\left(k_{d}d_{d}\right)-\left(1+{\text{α}}_{s,p}^{2}\right)\text{sin}\left(k_{m}d_{m}\right)\text{sin}\left(k_{d}d_{d}\right)/2{\text{α}}_{s,p}^{2}.$$

where

(2) $${\text{α}}_{s}=k_{d}/k_{m},$$

(3) $${\text{α}}_{p}=k_{d}{\text{ε}}_{m}/k_{m}{\text{ε}}_{d},$$

while the indices S and P are used to denote different polarizations.

(4) $$k_{m,d}={\left({\text{ε}}_{m,d}-k^{2}\right)}^{1/2}.$$

All spatial dimensions are normalized to c/ω, while the wavenumbers are normalized to ω/c.

If the thickness of the layers is subwavelength (the uni­axial crystal approximation), we may assume

(5) $$k_{m}d_{m}<<1.$$

(6) $$k_{d}d_{d}<<1$$

Further we introduce the effective dielectric permittivities for S and P polarization as

(7) $${\text{ε}}_{s}=\left(1-\text{δ}\right){\text{ε}}_{d}+\text{δ}{\text{ε}}_{m},$$

(8) $${\text{ε}}_{p}={\text{ε}}_{m}{\text{ε}}_{d}[\text{δ}{\text{ε}}_{d}+(1-\text{δ}){\text{ε}}_{m}],$$

where

(9) $$\text{δ}=d_{m}/L.$$

is the metal fill factor (the ratio between the metal stratum thickness and the total thickness of the unit cell).

For a general case, when there are three or more constituent materials in the planar multilayer, the expressions for the effective permittivity become [9]

(10) $${\text{ε}}^{\left(x,y\right)}=\sum_{i=1}^{N}{\text{δ}}_{i}{\text{ε}}_{i},$$

(11) $$\frac{1}{{\text{ε}}^{\left(z\right)}}=\sum_{i=1}^{N}\frac{{\text{δ}}_{i}}{{\text{ε}}_{i}},$$

where *N* is the number of constituent materials.

For the filling factor there is the obvious condition that

(12) $$\sum_{i=1}^{N}{\text{δ}}_{i}=1.$$

We may write the Floquet-Bloch dispersion relation for the subwavelength layer case as [112,115]

(13) $$(1/{\text{ε}}_{s}){\text{sin}}^{2}(qL/2)+(1/{\text{ε}}_{s,p})k^{2}{\left(L/2\right)}^{2}={\left(L/2\right)}^{2}.$$

One should bear in mind that although the effective medium theory is applied here, the above Floquet‑Bloch dispersion relation can be utilized without any limitations regarding the value of the product *qL*.

The standard way to calculate the dispersion of a finite 1D plasmonic crystal is to utilize the transfer matrix technique [132,133]. In our case it is

(14) $$\left(1/2\right)\left(T_{11}-T_{22}\right)\left(\text{sin}NqL\right)\left(\text{sin}qL\right)+\text{cos}NqL=0$$

where *T*_11_ and *T*_22_ are the diagonal elements of the transfer matrix to be calculated according to [132,133].

Further we consider two typical situations with symmetric geometries. One of them is when the cladding (the surrounding medium) is dielectric, while in the other case a metallic cladding is used. In both cases there is a metal-dielectric multilayer within.

**Figure 3:**
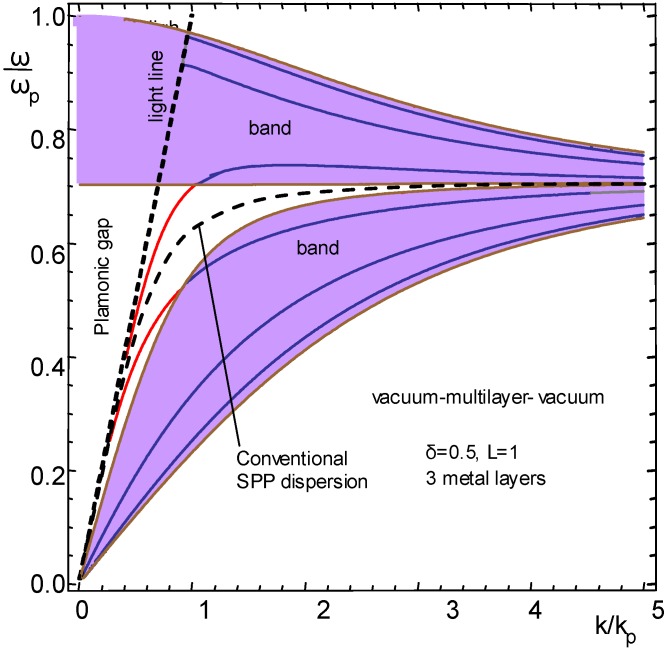
Dispersion of guided modes for *N* = 3, vacuum on both sides of the nanomembrane multilayer (exact solution).

Figure 3 shows the dispersion for a freestanding, self-supported structure with three metal layers, the surrounding medium being air or vacuum. In this configuration only P modes are supported.

It can be seen that two plasmonic bands appear (shaded) with a gap between them. Each band is split into three branches of P-modes, their number being equal to the number of metal strata. The dashed line in the middle of the gap represents the dispersion of the conventional SPP wave.

One can see that the lowest branch in the top band crosses from the band and enters the plasmonic band gap. Its value for high wavevectors tends asymptotically to the conventional SPP dispersion.

All branches in the top band decline for a certain range of values of the normalized wavevector, that is, the group velocity becomes negative. The problem for a practical implementation of such modes is that the effect appears near the frequency values close to ω/ω_p_ = 1, where losses are very large.

The highest branch in the bottom band also crosses between the plasmonic band and the band gap, similar to the lowest branch in the top band. None of the modes crosses the light line, meaning that their coupling with the propagating modes must remain like those used in conventional SPP devices.

**Figure 4:**
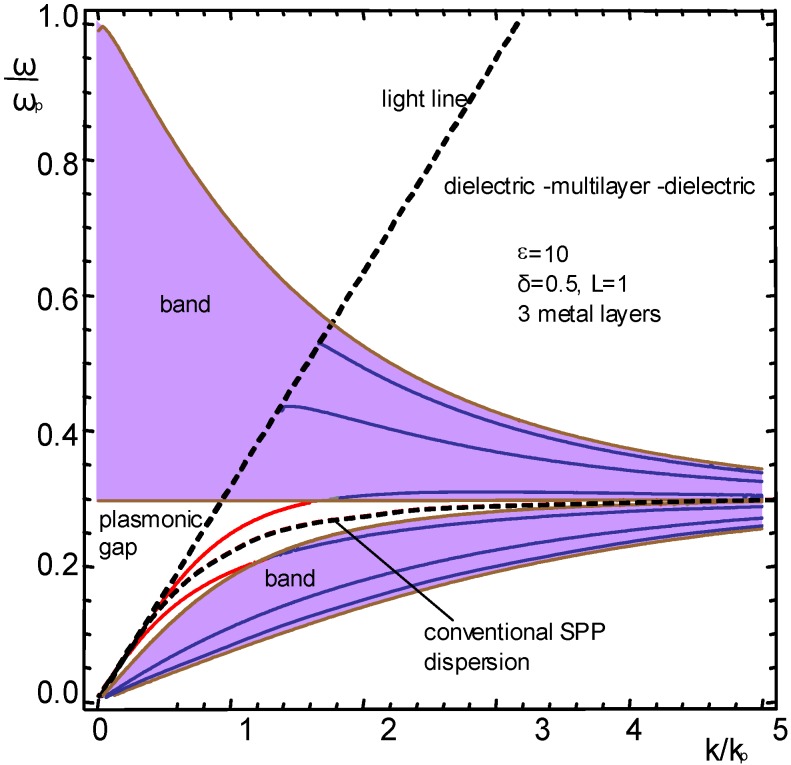
Dispersion of guided modes for N = 3, dielectric ε = 10 on both sides of the nanomembrane multilayer (exact solution).

A similar situation is shown in Figure 4, but with one important difference. All parameters of the SPC guide are identical, but the cladding is a medium with a larger dielectric permittivity (for instance, analyte fluid with high refractive index, or a guide immersed in a high-permittivity dielectric).

**Figure 5:**
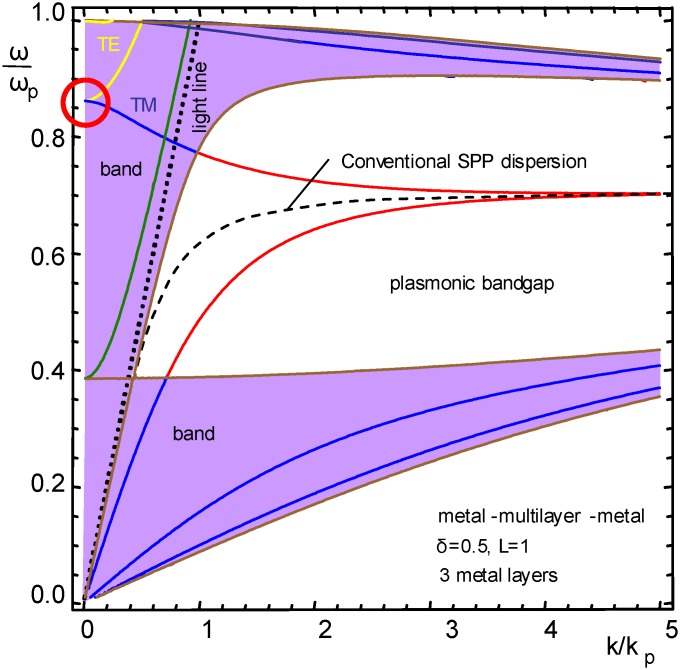
Dispersion of guided modes for *N* = 3, Drude metal on both sides of the nanomembrane multilayer (exact solution).

Qualitative behavior of the dispersion in this case is expectedly similar to the case of the 1D SPC in vacuum/air, but all P-mode branches are shifted towards lower frequencies. This means that in this case the losses in the negative group velocity range are much smaller.

A very interesting situation is encountered if the multilayer geometry and composition are kept, but the cladding is metal, as shown in Figure 5. This corresponds to a situation where a metal-cladded waveguide is used with a planar cavity, to which analytes may be introduced from the outside in a manner similar to that in photonic crystal fiber sensors.

S-modes appear in this situation, all of them within the light cone, in addition to the conventional P modes. The S band also has three branches, but only the lowest can be seen in Figure 5, while the other two cannot be discerned, being too close to the frequency of ω/ω_p_ = 1.

As in the previous case, 2*N* P-polarized modes appear, *N* in the upper and *N* in the lower band, with a plasmonic bandgap between them. The negative group velocity ranges again appear in the top band. The conventional SPP dispersion is once more the limiting value to which the bottom curve from the top band and the top curve of the bottom band tend. Both of these curves cross between the plasmonic bands and bandgap, but this time they start in the plasmonic bands at lower wavevectors (where the bands overlap) and a plasmonic bandgap to which they cross is formed only for higher wavevectors.

There is, however, an essential difference compared to the previous symmetric situations. The negative group velocity modes from the top plasmonic band cross the light line. Thus it becomes possible here to excite negative group velocity modes directly by the propagating waves.

A red circle denotes the point where one S and one P mode reach zero inclination for the zero-value of wavevector. This happens for the bottom-most branches of S and P modes in the top plasmonic band. These modes are actually Tamm plasmon polaritons [110,111].

It is worth noting that in situations when there is a single ultrathin freestanding metal layer surrounded on both sides by dielectric cladding (an experimental situation corresponding to this is described in [94]) we arrive to the well-known long-range SPP guide [107,134]. Another possibility to retain a single monolithic SPP layer geometry is to introduce periodic thickness modulation into such a structure, *i.e*., to fabricate Bragg grating from a freestanding membrane [135]. The long-range SPP chemical, biochemical or biological sensors which utilize this geometry are currently the most sensitive plasmonic sensors [136].

## 5. Metasurfaces Based on Split Ring and Double Split Ring Resonators—Case of Magnetic Metamaterials

**Figure 6:**
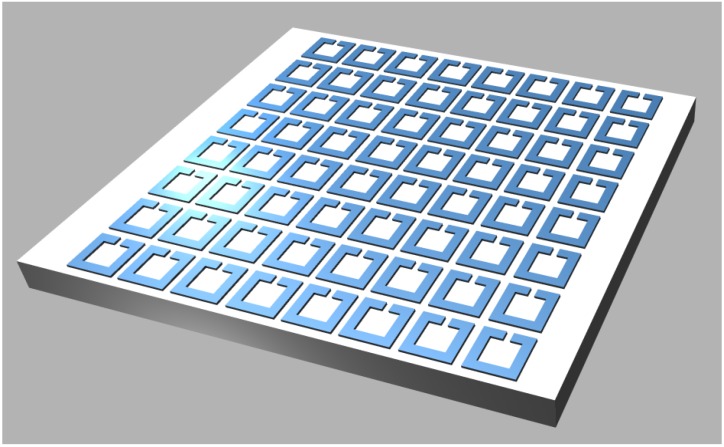
A prototype magnetic metasurface: an array of split ring resonators stacked in a square 2D lattice.

A more complex case of planar plasmonic crystals are 2D structures with a pattern repeating on the surface in a wallpaper fashion. An obvious way to obtain such surfaces is to utilize an electric or magnetic “atom” or “particle”—the planar unit cell furnishing either magnetic or electric response. One of such metamaterial “particles” is the split ring resonator (SRR). A SRR represents a nonmagnetic conductive ring structure with one or more narrow gaps (splits) filled with dielectric and possibly fabricated as two or more concentric contours. The shape of a split ring may be circular, square, hexagonal, triangular, or actually it may be any closed contour. Complementary structures to SRR include the corresponding shapes formed as slits in a continuous metal surface. Figure 6 shows a metasurface composed from a 2D array of single split rings on dielectric surface.

**Figure 7:**
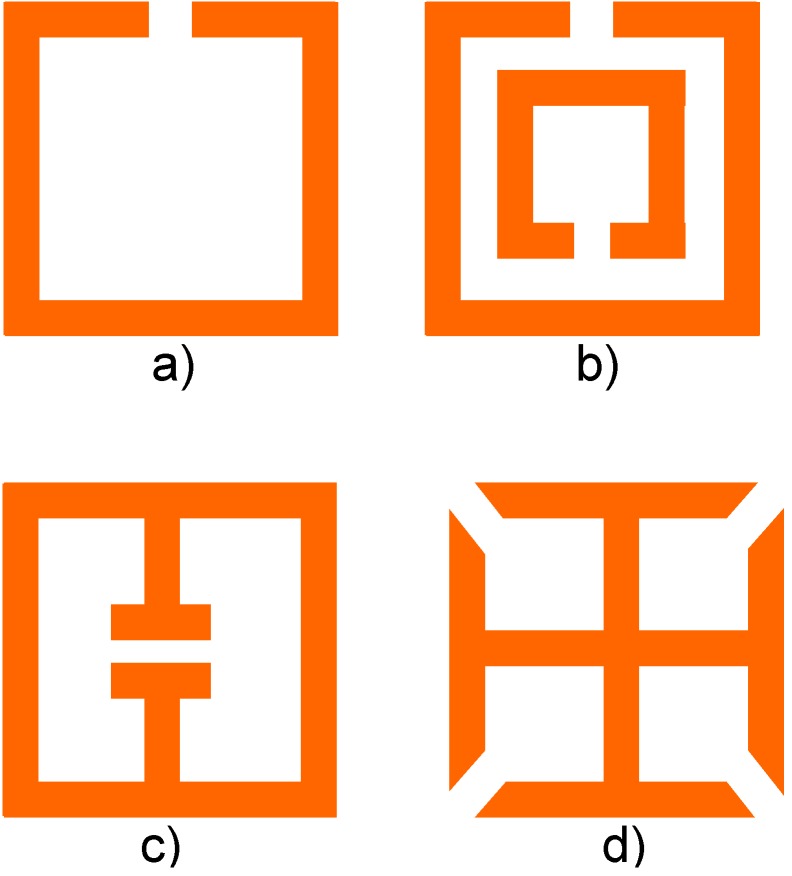
Some basic forms of split ring resonators with square unit cell.

Historically, SRR were the first magnetic “particle” furnishing negative effective magnetic permeability for metamaterials [8]. The SRR has since been used as a prototype meta-atom in a large body of theoretical and experimental papers. While the number of possible implementations is literally endless, Figure 7 shows some possible approaches.

A SRR actually represents a planar inductive-capacitive circuit. The conductive loop (or loops) of its rings ensures self-inductance. The dielectric gap determines the capacitance. Similar to other metamaterial “particles”, the dimensions of a SRR structure must be deeply in the subwavelength region. Since its capacitance and inductance determine the resonant frequency and at the same time define the shape of its spectral dispersion, one will be able to use their values to determine the effective relative permeability of the structure that consists of an array of SRRs.

The resonance frequency of an SRR element is determined according to the well-known dependence for an LC circuit

(15) $${\text{ω}}_{0}\sim \frac{1}{\sqrt{LC}}.$$

The capacitance of the element is on the other hand

(16) $$C_{SRR}=\frac{1}{{\text{ε}}_{0}\sqrt{\int_{V}\text{ε}(V)E(V)dV}}$$

where the volume *V* envelops the space above the SRR where the evanescent field is non-negligible.

The dispersion of their effective permeability is strongly resonant and may be described by the generic expression

(17) $$\text{μ}(\text{ω})=1-\frac{F{\text{ω}}^{2}}{{\text{ω}}^{2}-{\text{ω}}_{0}^{2}}$$

One should mention here that SRRs are magnetic metastructures, meaning that their magnetic response becomes weaker with increasing frequencies, finally vanishing at visible frequencies where the term of the permeability itself loses its meaning [137]. Already at frequencies above 100 THz the resonant frequency *versus* the resonator dimensions becomes saturated and the relative permeability decreases (depends on the geometry, however). At optical frequencies the effective permeability of split ring resonators does not reach negative value at all [138]. This is contributed to the increase of losses and the saturation of the resonance, resulting in increased electron scattering at the metasurface and the skin depth even exceeding the thickness of the metal part. Thus the majority of the experimental SRR planar metamaterials until now were designed for the microwave and terahertz frequency ranges. Still, there are experimental SRR designs that were intended for the infrared wavelengths [42], even for the visible range [139]. It has been shown that structures with a larger number of dielectric gaps exhibit a better high frequency performance [140].

The SRR-based metasurfaces were among the first experimental metamaterial surfaces proposed for chemical or biological sensing—the “wallpaper” design [79].

To detect chemical or biological analytes one makes use of the electromagnetic field enhancement within the dielectric gap of the split ring, near the surface of the metal parts of the SRR array or both. The presence of the analyte due to the array immersion into it or caused by adsorption will result in a change of the refractive index and thus tune the propagation conditions for the surface Plasmon polaritons.

Several authors described the use of experimental asymmetric split ring resonators for SRR-based chemical sensing. Debus and Bolivar presented the use of SRR surfaces with an analyte overlayer for the terahertz range [141]. Al-Naib *et al*. utilized rectangular asymmetric double SRR with tips for field confinement [142].

O’Hara and coworkers consider an experimental SRR wallpaper geometry with an analyte overlayer ranging in thickness from 0.1 nm to 16 µm [81]. The presence of the analyte changed the refractive index in the top stratum and thus shifted the resonant frequency

A similar scheme was described in [77] where the electromagnetic properties of SRR were modified by applying drops of silicon-nanospheres dispersed in ethanol. The presence of the analyte caused a shift of the magnetic resonance toward lower frequencies.

As mentioned above, one approach to SRR-based chemical or biological sensors is to bring the analyte near the gap. This was used by Bingham *et al*. [79] for their sensors intended for terahertz range. In their experiments they used different SRR designs, including superlattices formed by stacking SRRs of different size and shape into hexagonal and square unit cells. The superlattices included one, two or three different sub-lattices, where the filling fraction was kept at maximum.

The reason to use split ring resonator superlattices was that each sublattice furnished its resonant electromagnetic response at a different frequency. Thus the authors obtained a multi-frequency response and in that way expanded the operating frequency range. This ensured enhanced selectivity and fingerprinting of the desired analyte, since each of the array resonances was tunable to a resonant frequency of a different targeted analyte.

A similar approach was used by Ozbay’s group where Caglayan *et al*. [143] used microwave excitation and field enhancement near the single split ring gap to perform the model biotin-avidin bioassay. They obtained a 35 times faster and two orders of magnitude more sensitive response than by competing methods (room temperature ELISA test).

Lahiri *et al*. used circular asymmetric single split-ring resonators to obtain metamaterial biosensors for the mid-infrared range (3–5) μm [80]. They used their sensors to detect (30–215) nm thin films of Polymethyl methacrylate. Their asymmetric single split ring structures had arms of different lengths, and each length introduced a different resonant frequency. The results were a sharper reflectance dip and an improved sensitivity.

Since the SRR sensors depend on the permittivity change in the position of maximum electric field, it appears obvious that the substrate permittivity will strongly influence their response. In fact, approximately 90% of the SRR capacitance is defined by the substrate permittivity [79]. A proposed way to overcome this and thus increase sensitivity is to use a low permittivity substrate. We develop this further to the point where the substrate is completely removed and a patterned freestanding nanomembrane is utilized as the metasurface instead. In such a situation the relative influence of the ambiental changes of the refractive index is even larger. This is further enhanced by the fact that the area coming in contact with the analyte includes both the top and the bottom surface (contrary to the conventional SPP sensors where a semi-infinite substrate is assumed). Thus the effective surface for the contact with the analyte during immersion or for analyte adsorption is twice larger than in previously reported sensing metasurfaces.

Another possibility is to combine the metasurface with a layer of ultra-low permittivity material, like those reported, for example, in [25,26].

## 6. Fishnet Structures—Double Negative Metamaterials

**Figure 8:**
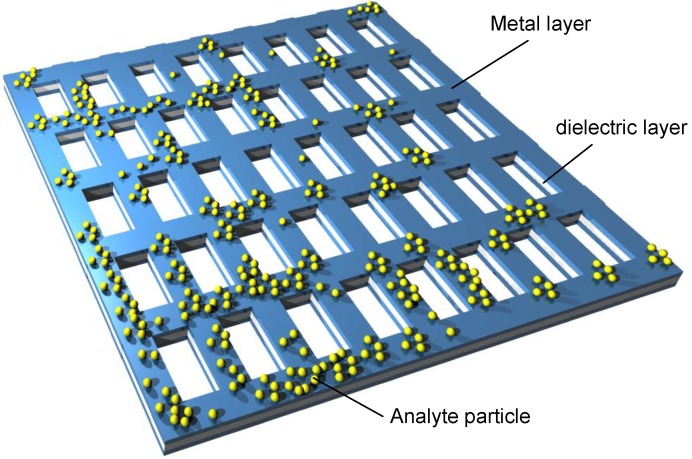
A segment of a double fishnet structure based on a freestanding nanomembrane; rectangular apertures in a square lattice.

**Figure 9:**
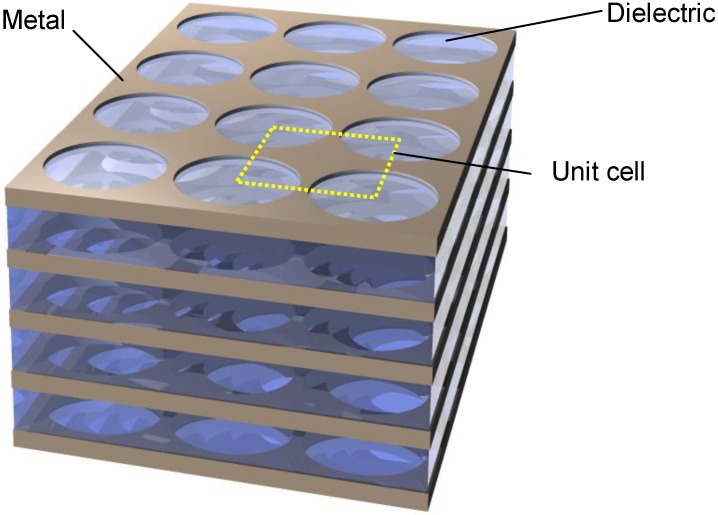
A multilayer stack of fishnet type, circular apertures filled with dielectric arranged in square lattice.

A unit cell of a fishnet NIM (a double fishnet) consists of two parallel metal sheets with a rectangular shape separated by a dielectric layer along the perpendicular direction. The sheets are discontinuous at each edge of the square. Each metal sheet is thus basically a superposition of a thin wire along one in-plane direction and a short slab along the other in-plane direction. If a large number of such unit cells are observed simultaneously, the structure has an appearance of a fishnet, where the discontinuities at the edges form the openings of the fishnet. These apertures may be, for example, rectangular, circular or ellipsoidal, or actually may assume any shape. An example of a fishnet with rectangular holes is shown in Figure 8. A generalization of this structure may include more alternating metal and dielectric layers [67]. A version of such a structure is shown in Figure 9.

Double-fishnet structures (also denoted as “double-grating” structures) are the negative index metamaterials for the optical range with the highest operating frequencies attained until now and with the best performance to date [42,144]. Their operating wavelengths even enter the visible spectrum [46,139]. Currently they are the prevailing structures for the optical wavelength range.

Among the advantages of the fishnet NIMs is their ability to generate left-handed response for a light beam incident perpendicular to the plane of the metal sheets. This was, for instance, handled experimentally in [145], where both normal and tilted incidences were considered in an experimental fishnet made of gold and TiO_2_ sandwich 30-50-30 nm (unit cell with dimensions 565 nm, 265 nm, and c = 860 nm, the main transmission peak in the spectral range 1.5–2.2 μm). In this way both lateral and normal coupling is ensured. Thus one is able to obtain a strong double‑negative response using a single metal-dielectric-metal structure.

**Figure 10:**
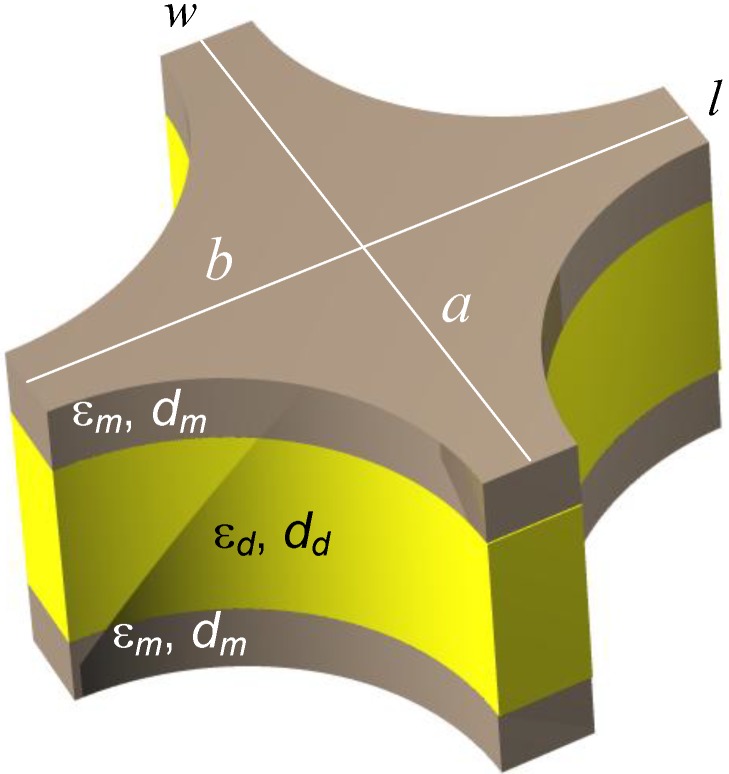
A unit cell of a double fishnet structure with circular apertures. The basic parameters of the structure are denoted in the picture. The permeability of the both constituents is equal to 1.

The geometry of a single unit cell of the fishnet MM with circular apertures is shown in Figure 10. The width and the length of the unit cell are a and b, respectively. The spacing between the metal sheets in Figure 10 generally may be filled with any dielectric (and actually with any material whatsoever) including air or the gaseous or liquid analyte.

**Figure 11:**
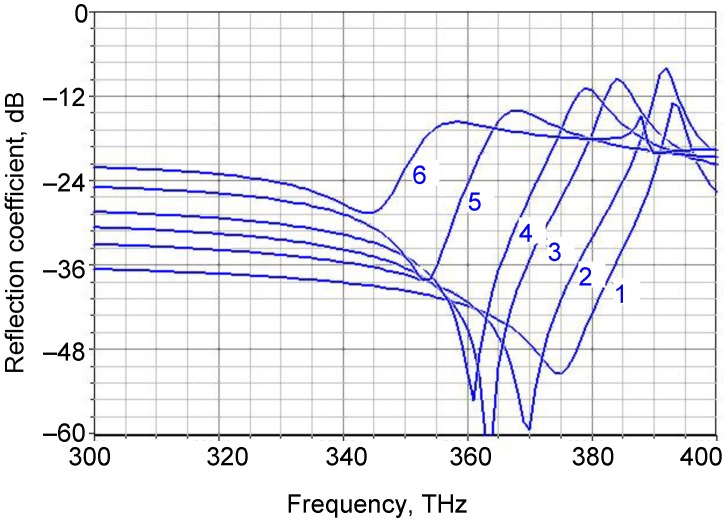
Spectral reflection for a fishnet PEC-dielectric-PEC with *d_m_* = 5 nm and various values of dielectric thickness, (1) 20 nm; (2) 50 nm; (3) 75 nm; (4) 100 nm; (5) 150 nm and (6) 200 nm. Relative permittivity of dielectric ε = 2.

Figure 11 shows the spectral reflection of a fishnet with a PEC-dielectric-PEC sandwich (PEC stands for Perfect Electric Conductor) where the metal thickness is *d_m_* = 5 nm. The dielectric thickness was varied as a parameter. The relative permittivity of the dielectric was always ε = 2. The frequency of the reflection dip shifts toward lower frequencies with the increasing thickness of the dielectric middle layer.

On the other hand, higher values of dielectric permittivity tend to shift the curves towards lower frequencies; thus it appears desirable to keep the difference between the real parts of dielectric permittivity between layers at its maximum. This situation, in what are essentially plasmonic crystals, is hardly surprising, since a similar condition occurs in the case of photonic crystals.

**Figure 12:**
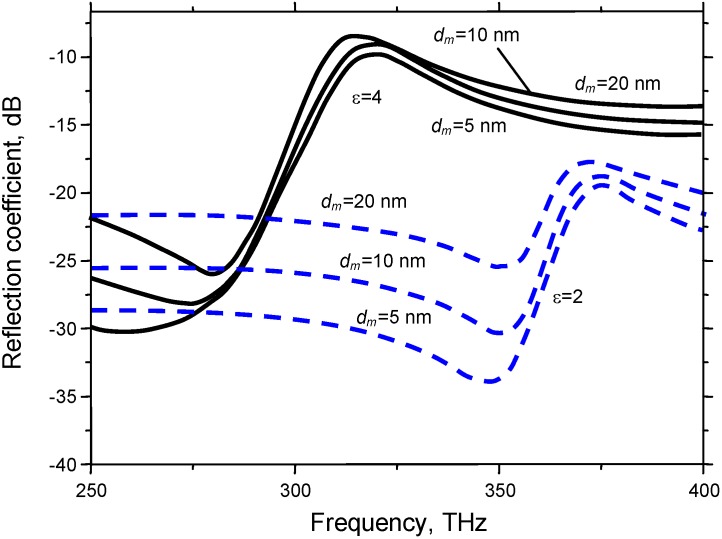
Spectral reflection for a silver-dielectric-silver fishnet for metal thickness of (1) 5 nm; (2) 10 nm; (3) 20 nm and a dielectric thickness of *d_d_* = 100 nm; ε_d_ = 2 or 4.

Figure 12 shows the influence of the silver strata thickness on the scattering parameters of the fishnet metasurfaces. The main change is an increase in the overall reflection coefficient with the increasing metal thickness. The frequency shifts, if any, remain relatively small. Absorptive losses, however, play an important role.

**Figure 13:**
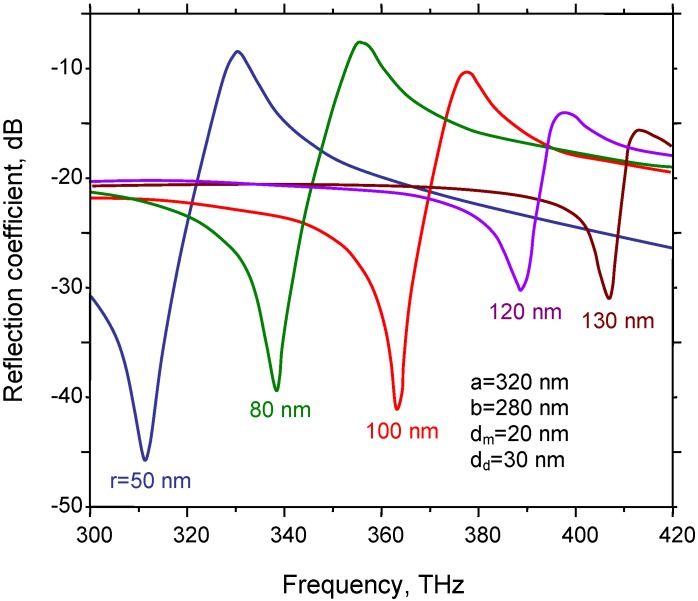
Influence of hole radius on the spectral reflection of the metal-dielectric-metal fishnet MM.

Another consideration of interest is that of the influence of the aperture size on the spectral characteristics. For this purpose the unit cell is kept constant (*a* = 320 nm, *b* = 280 nm) and the aperture radius is varied from 50 nm to 130 nm. As expected, the increase in the aperture radius strongly shifts the dependences toward higher frequencies (Figure 13).

**Figure 14:**
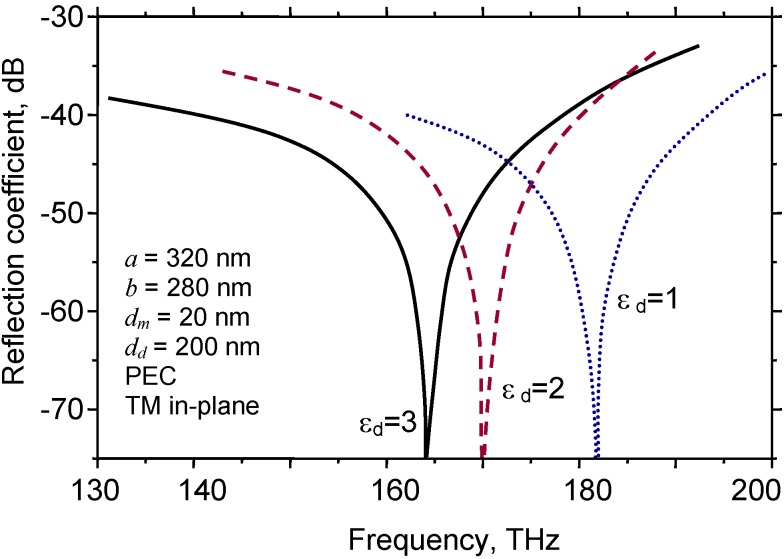
Reflection from a metal-dielectric-metal symmetric freestanding fishnet MM with circular holes. Geometrical dimensions and analyte properties are shown.

The most interesting consideration pertains to the influence of analytes on the scattering parameters of the fishnet. Figure 14 shows a situation where a fishnet unit cell is fully immersed into analyte. Such a situation may occur if the fishnet sample is used in a microfluidic scheme where the analyte flows through the apertures. Similar experimental sensing setups were described previously in a context of subwavelength hole arrays [83,85]. The electromagnetic response was calculated by the finite element method for a rectangular-array structure with a unit cell of *a* = 320 nm, *b* = 280 nm, perfect electric conductor. As expected, the presence of the analyte caused a spectral shift of the reflection dip. It can be seen that thinner and smaller structures are more strongly tuned by the presence of analyte.

**Figure 15:**
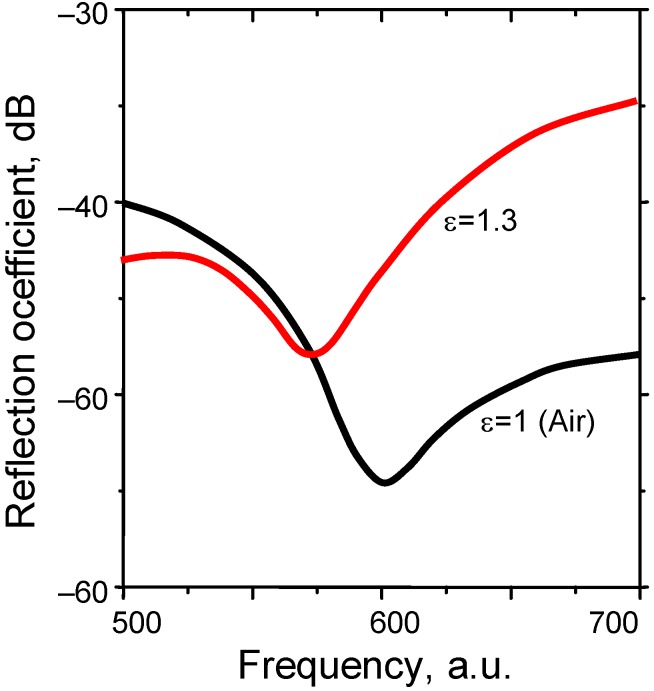
Reflection from a metal-dielectric-metal symmetric freestanding fishnet MM with circular holes in the case of PEC without adsorbed dielectric (surrounding ε = 1.0) and with an adlayer (ε = 1.3).

Another scheme occurs when only a thin adlayer of analyte (down to a monomolecular or monatomic thickness) is adsorbed to the fishnet metamaterial surface, analogous to conventional SPP sensors. Figure 15 shows a situation where a circular aperture array is used with a unit cell *d* = 300 nm, and the plasmonic part is a perfect electric conductor. The analyte adsorption causes a reflection dip shift towards lower frequencies in a manner similar to that in conventional SPP devices.

## 7. Metasurface Multifunctionalization for CBB Sensing

Functionalization of ultrathin films that comprise metasurfaces can be performed in a variety of ways [146]. These include nanocompositing with fillers, lamination, additive or subtractive patterning and surface sculpting. Actually the very formation of wallpaper-type single-negative surfaces belongs to the class of additive patterning, while 1D plasmonic crystals belong to the class of laminated nanocomposites. Since such an approach ensures the possibility to impart two or more parallel roles to the structure, they belong to the class of multifunctionalization.

In this section we consider two groups of functionalization which both utilize multiple functions: one of them is CBB sensor selectivity enhancement through integration with various membrane-based filters and the other is bioassaying through dual patterning.

In the organic and biological world many analytes have very similar or identical values of refractive index, so that the question of selectivity is very important to plasmonic sensors which measure only the refractive index changes. The conventional approach in SPP sensors is to utilize some kind of ligand layer on the plasmonic surface which is only able to bind with a specific analyte [1]. The method is well developed and efficient and is also fully applicable to generalized metamaterial-based sensors. However, the geometry of metasurfaces and especially the use of freestanding films ensure an additional degree of freedom in their selectivity enhancement.

An obvious way is to use nanomembranes as additional filters for molecular separation. They could be laminated with the metasurfaces as an additional layer or fully integrated, could be combined with a ligand layer, or used separately. Actually molecular separation belongs to the most basic applications of both conventional membranes and artificial nanomembranes [147,148,149].

Compared to the conventional membranes, nanomembranes are typically at least 2–3 orders of magnitude thinner (tens of nanometers compared to tens or even hundreds of micrometers). Because of this they have much shorter paths for analyte particles and thus higher throughputs, resulting in the separation process being much faster. This means that a nanomembrane filter will generally not compromise the overall speed of an integrated metasurface CBB sensor. Another advantage of using nanomembrane separators compared to conventional membranes, is that it is easier to retain the desired nanopore aspect ratio in a structure with a nanometer thickness than in a micrometer one. One result of this improved accuracy is that the selectivity of nanomembrane-based filters is additionally enhanced compared to conventional structures. Indeed, nanopores are utilized in, for example, DNA and RNA characterization [150] and even for the differentiation between molecules which only differ by a single nucleotide [151].

Here we quote four different approaches to membrane-enhanced selectivity [152] that can be applied for metasurface-based plasmon sensors.

(1) Pore-based molecular sieves. Separation between different molecules is done by their passage through randomly distributed and interconnected macro-, meso- or micropores (a particle filter approach). Larger molecules are rejected, while all smaller particles pass the nanomembrane. The pore distribution itself may be isotropic or anisotropic.

(2) Ion exchangers. Here the membrane (typically nanoporous) contains positive or negative ions incorporated in the pore walls. These ions bind the oppositely charged analyte particles.

(3) Gated ion channels. This is an approach utilized solely in nanomembranes, and typically in lipid bilayer organic membranes like those forming the walls of the eukaryotic cells, although various different materials may also be used [146]. The channel is gated by an external stimulus, for instance by applied voltage or by ligands, which open or close the gate for ion transport, resulting in extremely high selectivity. Various ion channels exist which are permeable to, for example, sodium, potassium, and hydrogen ions only. For instance, aquaporin proteins permit the flow of water molecules but are impermeable to smaller protons, while Grotthuss “water wires” do the opposite by conducting protons and blocking other particles [153].

(4) Dense membranes with solution-diffusion mechanism. In this case there are no pores, but the analyte transport proceeds as a combination of particles solution and their further diffusion. The minute thickness of nanomembranes in this case is again greatly beneficial in increasing the overall speed of the process.

Bioassaying through dual patterning is another approach to improve selectivity of CBB sensors. It is connected solely with the metamaterial-based and nanoplasmonic devices. The idea is to utilize metasurface patterning in a dual way. The structure at the same time serves as a 2D subwavelength plasmonic crystal and the pattern/material combination type which attracts certain type of bioanalytes, for instance the specific kind of bacteria or organic tissue. It is known that various kinds of patterning of the substrate may promote adhesion of living cells, from bacteria to animal and human tissue cells [154,155,156,157]. By performing engineering of the pattern to ensure the desired electromagnetic response (for instance, single- or double-negative behavior) while simultaneously retaining selective adhesion towards certain types of microorganisms, one may obtain structures which preferentially attract the targeted bioanalytes without any adhesive protein buffer layers.

## 8. Intrinsic Noise Sources

The performance of generalized plasmon sensors based on metasurfaces is limited by fluctuation phenomena which may be caused by various external and internal mechanisms and which result in noise appearing in the sensor readout. This is the same situation as with practically any other sensor. In this section we give a short review of the most important intrinsic mechanisms of noise which are connected with the detection mechanism itself and which cause stochastic fluctuations of the measured refractive index at the metasurface. Most of these mechanisms are fundamentally related with the detection process.

External noise sources related with plasmonic surfaces [136] include, for example, quantum noise/shot noise of the light source [158]), photodetector noise (generation-recombina-tion, 1/f and Johnson-Nyquist noise [159]) and noise of the processing circuitry.

In the case of a plasmonic sensor, intrinsic noise is defined by the stochastic temporal and spatial fluctuations of the refractive index at the active surface-analyte interface which are “seen” by the surface plasmon wave.

As far as the intrinsic sources of noise appearing in metasurfaces are concerned, there are three main contributing mechanisms [160]. The first is identical to the processes occurring with conventional SPP devices and is connected with the adsorption and desorption (a–d) of analyte particles on the metasurface. Similar to the generation-recombination noise in photodetectors, this is a fluctuation mechanism connected with the detection process itself and cannot be separated from it [161].

Another fundamental noise mechanism is Johnson-Nyquist (thermal) noise, *i.e*., thermal fluctuations of the refractive index at the metasurface-adsorbate interface [160]. Thermal effects are also felt as the fluctuations of the blackbody radiation and as zero-point (vacuum) fluctuations [162]. The latter are especially important if the analyte is of a nonlinear substance with polar molecules, as is the case with many organic analytes.

Finally, there is 1/f (flicker) noise, connected with traps and imperfections in sensor material. Although not fundamental, like a–d noise, it is omnipresent and must be taken into account in realistic situations.

The a–d noise has been investigated by Yong and Vig *et al.* [163,164] for the case of microelectromechanical (MEMS) resonators and by Jaksic *et al*. [13,160] for plasmonic sensors. On metasurfaces, analyte particles will adsorb onto them or desorb, modifying the effective refractive index at the interface and causing its fluctuations.

In case of physisorption of a single gaseous analyte on the metasurface which forms a monolayer, the change of the refractive index at the surface will be determined by the fluctuations of the number of adsorbed and desorbed particles of the analyte, which is given by an ordinary differential equation of the form

(18) $$\frac{d(\Delta N)}{dt}=v_{ad}(N)-v_{de}(N)$$

where *v_ad_*(*N*) and *v_d_*_e_(*N*) are the adsorption and desorption rates.

The solution for the number of particles is

(19) $$\Delta N_{S}^{2}(f)=\frac{4C_{2}N_{\text{max}}{\text{θ}}_{0}A{\text{τ}}^{2}}{1+4{\text{π}}^{2}f^{2}{\text{τ}}^{2}}$$

where *A* is the active adsorbing area, *f* is frequency, *N_max_* is the maximum number of adsorbable particles per unit area, and θ is the surface coverage. The time during which the system reaches a stationary state is

(20) $$\text{τ}=\frac{{\text{τ}}_{0}\text{exp}\left(E_{d}/RT\right)}{bp+1}$$

where τ_0_ is the period of thermal vibrations of an adsorbed particle perpendicular to the surface. *E_d_* is the desorption energy, *R* is the gas constant, *p* is the partial pressure of the analyte gas, and *b* = α_s_*C*_1_<τ>/*N*_max_ , while α_s_ is the sticking coefficient.

The effective refractive index of the adsorbed monolayer is calculated utilizing the simple mixing rule

(21) $$n=n_{eff}=fn_{a}+(1-f)n_{e}$$

where *n_a_* is the refractive index of the analyte, and *n_e_* is the refractive index of the environment, *f* = *V_AD_*/*V_max_* is the ratio between the adsorbed and maximal adsorbable analyte volume

(22) $$n=n_{e}+\frac{N_{ad}}{N_{m}A}[n_{a}-n_{e}]$$

and its change due to fluctuations is

(23) $$\Delta n_{ad}=\frac{\text{δ}N_{ad}}{N_{m}A}[n_{a}-n_{e}],$$

The spectral power density of refractive index fluctuations is [160,161]

(24) $$\begin{array}{l} \bar{{\left(\Delta n(f)\right)}^{2}}={\left(\frac{n_{e}-n_{A}}{N_{\text{max}}A}\right)}^{2}\;\bar{\Delta N^{2}(f)}\\ =4{\left(\frac{n_{e}-n_{A}}{N_{\text{max}}A}\right)}^{2}\frac{C_{2}N_{\text{max}}bpA}{C_{2}^{2}{\left(bp+1\right)}^{3}+4\text{π}f^{2}\left(1+bp\right)} \end{array}$$

It is important to note that the above equation is applicable both in the case of positive and of negative values of refractive index. The above procedure may be applied in an analogous manner to calculate the effective permeability or permittivity.

An important result obtained when calculating a–d noise at nanoplasmonic surfaces is that it depends on the active area of the metasurfaces and is higher for smaller areas. This is intuitively expected: if an area is smaller, then the same number of adsorbed (or desorbed) molecules will introduce a higher relative change and thus higher noise.

Thermal fluctuations (Johnson-Nyquist noise) may be connected with the Brownian motion of free electrons in the metal part and with the thermal oscillations of atoms/molecules in both metal and dielectric parts.

In a general case the spectral density of thermal noise voltage is

(25) $$\langle \text{δ}V^{2}\left(\text{ω}\right)\rangle =\frac{2}{\text{π}}\int_{0}^{\infty }R(\text{ω})\left(\frac{\hbar \text{ω}}{2}+\frac{\hbar \text{ω}}{\text{exp}(\hbar \text{ω}/kT)-1}\right)d\text{ω}$$

where $\underset{\cdot }{R}(\underset{\cdot }{\text{ω}})$ is the Ohmic resistance. The left term in the bracket describes quantum noise—the zero-point energy of the free electrons (vacuum fluctuations), and the right term is the thermal component and describes field fluctuations in blackbody radiation.

In the case *h* $\underset{\cdot }{\text{ν}}$ << *kT* and for a dispersionless electrical conductivity

(26) $$\langle \text{δ}V^{2}\rangle =4\text{π}kTR\Delta f$$

the well-known expression in semiconductor devices.

Another part of Johnson-Nyquist noise at metasurfaces are thermal fluctuations of ions (valid both for the metal and dielectric part). These phonon fluctuations result in fluctuations of the refractive index, as described by Glenn [165]. The thermodynamic state of a system is characterized by its density ρ and temperature *T* so that the fluctuations of refractive index are

(27) $$\Delta n={\left(\frac{\partial n}{\partial \text{ρ}}\right)}_{T}\Delta \text{ρ}+{\left(\frac{\partial n}{\partial T}\right)}_{\text{ρ}}\Delta T$$

The spectral power density of effective refractive index fluctuations is

(28) $${<\Delta n^{2}>}_{opt\;therm}={\left(\frac{\partial n}{\partial \text{ρ}}\right)}_{T}^{2}<\Delta {\text{ρ}}^{2}>+{\left(\frac{\partial n}{\partial T}\right)}_{\text{ρ}}^{2}<\Delta T^{2}>$$

or

(29) $${<\Delta n^{2}>}_{opt\;therm}=\frac{kT{\text{ρ}}^{2}}{V^{2}}{\left(\frac{\partial V}{\partial p}\right)}_{T}{\left(\frac{\partial n}{\partial \text{ρ}}\right)}_{T}^{2}+\frac{kT^{2}}{\text{ρ}VC}{\left(\frac{\partial n}{\partial T}\right)}_{T}{\left(\frac{\partial n}{\partial T}\right)}_{\text{ρ}}^{2}$$

where *C* is the specific heat capacity and *V* is the system volume.

At metasurfaces at optical frequencies electron fluctuations should prevail over the phonon ones.

The 1/f spectrum in refractive index fluctuations was predicted in [166]. The expression for the spectral power density due to 1/f noise has the well-known form

(30) $${<\Delta n^{2}>}_{1/f}=\text{ξ}/{\text{ω}}^{\gamma }$$

where ξ and γ are parameters connected with the particular material.

If we assume that the individual noise mechanisms are mutually independent, the total intrinsic noise in a metasurface-based generalized plasmon will be

(31) $${<\Delta n^{2}>}_{tot}={<\Delta n^{2}>}_{opt\;therm}+{<\Delta n^{2}>}_{AD}+{<\Delta n^{2}>}_{1/f}$$

## 9. Conclusions

In this paper we have considered theoretical and experimental approaches to the use of some planar electromagnetic metamaterials (metasurfaces) in plasmon-based chemical, biochemical or biological sensing. Most of the contemporary experiments with metamaterial-based CBB sensors are limited to planar structures, which is a natural consequence of the fact that they are the easiest to fabricate utilizing the state-of-the-art micro- and nanofabrication technologies.

Although many phenomena and structures connected with the NIM can be used for CBB sensing, in this work we limited ourselves to only the few most important ones. Even the simplest structures considered here, the 1D subwavelength plasmonic crystals (laminar ultrathin films which may be freestanding or not) exhibit a rich variety of electromagnetic modes, including those with negative and near-zero group velocity, which could be used for chemical or biological sensing. The possibility to use the novel plasmonic scaffold with fully symmetric geometry, the freestanding or free-floating nanomembranes, adds an additional degree of freedom to an already vast field. The further possibility of directly combining metasurfaces-based sensors both with optical and electronic circuitry also offers a great advantage.

Obviously, the field of generalized plasmon sensors is only beginning to develop; the first eligible mechanisms being described, and the first sensors being proposed, only a few years ago. This manuscript outlines the first steps taken and indicates some possible directions for future investigations. As is usually the case with novel fields of research, some prospective approaches may be abandoned and completely new ideas and approaches may, and probably will, appear, but generally it seems that the metamaterial-based CBB sensors as a whole can expect a bright future.
